# A grounded theory study exploring immigrant Muslim women’s perceptions and experiences of menopause, hormone replacement therapy and menopause-related healthcare in the UK

**DOI:** 10.1177/20533691251322823

**Published:** 2025-03-02

**Authors:** Reemanne Baghdadi, Neil Singh, Anne Gatuguta

**Affiliations:** 1 Primary Care and Public Health, 12190 Brighton and Sussex Medical School, Brighton, UK; 2 1948 University of Sussex, Brighton, UK; 3 1947 University of Brighton, Brighton, UK; 4 Global Health, 12190 Brighton and Sussex Medical School, Brighton, UK

**Keywords:** Menopause, perimenopause, Muslim women, experiences, perceptions, hormone replacement therapy, menopause-related healthcare, migrant women

## Abstract

**Aim:**

Menopause, a significant life transition affecting all women, is underexplored among minoritised groups. No United Kingdom (UK) based studies have specifically investigated Muslim women’s menopause experiences. This study examines immigrant Muslim women’s experiences of and perspectives on menopause, hormone replacement therapy (HRT), and menopause-related healthcare (MRH).

**Methods:**

Using constructivist grounded theory, we conducted online, in-depth, semi-structured interviews with participants recruited through the Brighton and Hove Muslim Women’s group. The interviews were audio-recorded, transcribed, and analysed through grounded theory coding (open, focused, selective, theoretical).

**Results:**

Twelve interviews revealed a core theme, ‘Regaining Agency’, shaped by three interconnected categories: ‘Losing Control of My Body’, ‘Dealing with Menopause Alone’, and ‘Navigating a Web of Silence’. Women reported physical and emotional challenges, including sleep disruption, cognitive changes, and anxiety, with cultural stigma and limited knowledge compounding negative perceptions and experiences. Health-seeking behaviours varied; while some women viewed menopause as natural, others saw it as a medical issue. Misconceptions about HRT led to hesitancy; however, users reported significant symptom relief. Many encountered barriers to MRH, often facing dismissive and uninformed healthcare providers.

**Conclusion:**

Enhanced education and support for menopausal women, along with mandatory training for general practitioners, are essential to improve the experience of menopausal transition for immigrant Muslim women.

## Introduction

Menopause is a universal transition that affects all women, and its significance grows as global demographics shift. By 2030, over one billion women are expected to be peri-menopausal or post-menopausal worldwide.^1^ Menopause, characterised by hormonal changes and the permanent end of menstruation, is often viewed through a biomedical lens as a biological decline.^2^ Symptoms such as irregular periods, hot flashes, depression, anxiety, cognitive changes, and sleep disturbances affect over 75% of women, significantly impacting quality of life.^3^

However, menopause is not only a biological experience; it is shaped by sociocultural factors and individual narratives.^2^ These influences affect how women perceive and manage their symptoms, seek support, and experience menopause-related healthcare.^4^ Currently, healthcare support for menopause often falls short of women’s needs, impacting their overall experience of this transition.^5,6^

While research increasingly recognises menopause as a complex, individualised experience, there is limited exploration of how sociocultural factors, perceptions, and healthcare access shape this process. Minoritised groups are particularly underrepresented, and in the UK, no studies have specifically explored the menopausal experiences of immigrant Muslim women. Yet, there is evidence that Muslim women in the UK have distinct health-related experiences^23^.

This study addresses this gap by exploring the nuanced experiences of Muslim women. It investigates four objectives: Muslim women’s experiences and attitudes towards menopause, their health-seeking behaviours and self-care practices, their knowledge of and attitudes toward hormone replacement therapy (HRT), their experiences with menopause-related healthcare.

Although menopause can affect individuals across gender identities, this study uses ‘women’ to refer to those assigned female at birth and does not include perspectives of trans men or non-binary individuals.

## Methods

### Participant recruitment

Purposive sampling was carried out, targeting self-confessed Muslim women, over the age of 40 who born outside of the UK and residing in Brighton and Hove (Table 1). In addition, participants had to be proficient in either English or Arabic. Women were recruited through the Brighton and Hove Muslim women’s group. Permission to recruit through the group was gained through a gatekeeper, who was the organiser of the group. Sample size was predetermined as 10–15 participants. However, a characteristic of Grounded Theory is concurrent collection and analysis of data. Which meant we recruited two participants at a time, conducted interviews, and analysed transcripts. Based on the data, new questions were asked of participants to elaborate and fill gaps in the emerging theory. More participants were recruited until theoretical saturation was reached. Saturation was reached after interviewing 12 participants.

### Data collection

To fulfil the research objectives, semi-structured 1:1 interviews with open ended questions were conducted. Interview guide was based on four categories: 1. Experience of, knowledge of, and attitudes towards menopause 2. Menopause-related health-seeking behaviours and self-care 3. Knowledge of and attitudes towards HRT 4. Experience of menopause-related healthcare (Table 2). Participant demographics were collected (Table 3). Interviews were in-depth eliciting each participant’s interpretation of their personal experience. Once eligibility had been established interviews were scheduled and conducted virtually using Skype and Microsoft Teams. Interviews were audio-recorded with permission and transcribed.

### Analysis

The initial analytical step involved reading through an entire transcript and noting down emergent themes. Analysis commenced with line-by-line coding, each line was labelled individually with a code that described an action or meaning (experience or perception). Codes used gerunds (‘-ing’ words) to indicate activity within the data. The following analysis process involved assessing the data on a more abstract level. Through a process of constant comparison, comparing all interviews and open codes, the open codes were organised into focused codes. Subsequently focused codes were organised into sub-categories and categories through selective coding and relationships between categories were determined through theoretical coding. An overall category was identified which linked to each category (relationship diagram, Figure 1). Throughout collecting and analysing the data, thoughts and ideas of connections, themes and patterns were captured through memo writing (Appendix 1) which aided in forming categories Appendix 2.

### Ethics

This study has been approved under the procedures of Brighton and Sussex Medical School (reference ER/BSMS9WK6/2).

## Results

### Category 1 – Losing control of my body

Women described the impact of menopausal changes on their lives, expressing lack of control (Tables 2, 4). Women noted uncontrollable and unmanageable changes to their appearance and overall wellbeing, changes included drier skin and changing hair texture, weight gain, visibly ageing and vasomotor symptoms. Women felt confused when reaching menopause and did not understand what was happening to their bodies. Some women never conceptualised menopause before reaching it, feeling that menopause had crept up on them. Some women attributed the confusion to lack of certainty and not knowing where to place themselves within the transition due to lack of confirmation from a healthcare professional. Women felt burdened by physical and psychological changes that accompanied menopausal transition. Women discussed changes in cognition and the ways in which their menopausal symptoms negetively impacted their ability to carry out their responsibilities at work. Women explained experiencing psychological changes ranging from minimal mood swings to more serious depression and anxiety.

### Category 2 – Dealing with menopause alone

Participants reported not receiving adequate MRH and expressed frustration of not being heard and understood (Tables 3, 5). Experiences of MRH were traumatising and caused unnecessary suffering. Participants explained refusal from GPs to prescribe HRT and their battle to receive treatment, this included severe and impactful symptoms being overlooked. GPs were uneducated on HRT options and when HRT was in fact prescribed, there was no discussion of alternative options or implementation of a care plan. Participants discussed what can be assumed to be systematic issues within the healthcare system.

Participants who did not seek out MRH noted a variety of reasons (Table 5). Doing their own research equipped them with enough knowledge to manage their symptoms, they did not deem symptoms severe enough to warrant consulting a healthcare professional (HCP) and some women were unaware of the availability of treatment options. Participants’ attitudes towards the beliefs surrounding the naturalness of menopause were presented in multiple contexts, affecting how they perceived menopause and HRT and how they accessed MRH. Participants expressed frustration about the expectation placed on women to cope with menopause alone and the naturalness of menopause being used to dismiss real struggles women experience. Conversely, normalisation has been expressed by some participants positively, they perceive menopause as a natural occurrence that they can handle and opposed medicalisation.

### Category 3 – Navigating a web of silence

Participants had limited understanding of menopause (Tables 4, 6). Participants explained that even when experiencing menopausal symptoms, they did not realise it was due to menopause. Participants attributed their limited knowledge to lack of discussion about women’s health and menopause. Participants spoke of societal attitudes towards the female body, explaining that whilst growing up women’s health was considered to be a taboo subject that is private and embarrassing. Some participants explained that they feel uncomfortable talking about menopause and do not want others knowing that they are menopausal. Attitudes were attributed to cultural traditions hindering the conversation of women’s health. Participants spoke of how negative terminology is associated with menopause and explained the cultural emphasis placed on reproductivity.

### Category 4 – Regaining agency

All women perceived menopause as simply another part of a woman’s life (Tables 5, 7). Participants explained that change in reproductivity does not deduct from their identity or sense of womanhood. Some participants perceived menopause as a time of liberation, freedom from the constraints of being a woman such as menstruation. Participants sought out information and took a proactive approach in their healthcare, recalling times in which they advocated for themselves during medical consultations. Participants made commitments to improve their health and wellbeing through making lifestyle changes. Women distinguished themselves from female relatives from previous generations by recognising accessibility and availability of information. Women explained how they attempted to break down generational cycles of silence by starting the conversation of women’s health within and outside the home, noting the importance of sharing information with their children to promote awareness and positive attitudes towards women’s health. Women described using humour when sharing their menopausal experience with others, reporting that this approach effectively broke down taboos and created a supportive environment. Participants noticed a perspective shift on menopause among the wider community and media in which other women are advocating for change to promote understanding and empowerment.

## Discussion

The core category in this study was regaining agency over menopause. Lack of agency was presented in the three other categories; lack of control over the body, insufficient information and support to manage changes and symptoms, and lack of control over the narrative of menopause. All participants reported efforts to gain control in one or more of these areas. The implications of these categories are discussed in this section.

Menopause profoundly impacted participant’s lives, consistent with the literature, including vasomotor symptoms,^7–9^ physical appearance changes,^10^ disturbed sleep,^9^ cognitive issues,^11,12^ and increased anxiety^8,9,12^ and depression.^7,11^ affecting their psychological wellbeing and workplace productivity. Previous studies have determined that the reported symptoms have negative affect on QoL,^13,14^ productivity and economic outcomes.^3^ Participants reported distress over bodily changes, which they had limited control over and were unprepared to manage, aligning with findings of unpredictability and unpreparedness.^5,6,10^ We can infer based on previous finding that a lack of knowledge and preparedness reported our participants may make the experience more taxing, physically and mentally, and can compound the negative impact of symptoms on QoL.^14–17^ Supporting this, participants that reported positive experiences also reported that they had prior knowledge of menopause and felt prepared. Participants emphasised the importance of formal education, including its integration in school curriculums and GP consultations.^5,6^

Participants reported negative, sometimes traumatic experiences of MRH, citing difficulty receiving appropriate HRT, feeling unheard by GPs and negative encounters with HCPs. This aligns with Aljumah’s findings, suggesting that this experience is shared across ethnic groups, as shown by similar reports from predominantly white British participants..^5^ Participants reported that a lack of diagnosis caused uncertainty, likely due to the medical community’s limited clarity on peri-menopausal symptoms.^18^ A UK study on GPs’ confidence in providing MRH reported a need for menopause training, suggesting that negative patient experiences may stem from educational gaps.^19^ These findings indicate MRH falls short of BMS and WHC recommendation,^20^ challenging parliament’s decision not to mandate menopause training for GPs.^21^

Participant’s health-seeking behaviours depended on symptom severity. Some believed their symptoms, though impactful, did not warrant medical intervention.^4^ Some participants were unaware of HRT,^22^ indicating unawareness of intervention. These participants were interviewed in Arabic, pointing to language barriers that hinder positive healthcare experiences for immigrant Muslim women.^23,24^ Some participants expressed frustration with the expectation to manage menopause independently, perceiving this as rooted in beliefs about menopause being ‘natural' and to be endured,^5^ and believed that HCPs held this view. This belief dismisses women’s concerns, causing suffering from inadequate support. Others felt empowered managing menopause naturally, rejecting HRT over side effect concerns.^6^ Conversely, participants who opted for HRT had positive experiences. Although menopause is not a disease and may be manageable, normalising it may be problematic due to long-term risks requiring prevention.^13^

Women discussed how menopause intersects with stigma around female sexuality and ageing, making it taboo. Cultural silence around menopause limited their knowledge,^25–27^ and societal framing of menopause as a ‘time of loss’ further complicates discussion. Ussher similarly reported that some women viewed menopause as the ‘age of despair’.^28^ However, our participants recognised this as inaccurate, separating their identity from reproductive status. The discrepancy may relate to average migration time- 6.3 years in Ussher’s study versus 28 in ours. Women noted differing attitudes toward menopause between their culture of origin and the UK's more accepting view. Migration may help some women reposition their views,^29^ though others retain original perspectives.^18^ Notably, ten participants completed higher education and nine were professionals, likely fostering identity beyond biological changes.^30^ Negative views of menopause, often linked to silence, may cause hesitancy in seeking advice due to stigma.

Women in our study viewed menopause as a time to gain control, marking a liberation from menstruation,^10,31^ allowing more freedom to practise religion, such as fasting and praying.^31^ Unlike Sergeant’s findings, which noted apprehension about losing religious and household task exemptions.^10^ Dissatisfied with MRH, participants took charge by researching treatments and sharing findings with their GP, while some successfully advocated for treatment that alleviated their symptoms and improved their lives, this may not be universal, highlighting the need for well-trained HCPs. Menopause also prompted awareness of age-related health risks, leading to holistic lifestyle changes.^10,26^ To break the silence, women initiated conversations.^5,6^ Some women in our study openly discussed their symptoms with colleagues and even strangers, this may be a coping mechanism that allowed them to gain understanding. In contrast, Sergeant found that most women kept menopause hidden, sharing only with friends and family.^10^ Most importantly, participants aimed empower their daughters with knowledge to reduce shame and stigma.

### Limitations

The sample is unrepresentative, as 10 of 12 participants have higher education (including 6 postgraduates), and 9 are professionals. Interviews were only in English and Arabic, potentially excluding non-fluent speakers. Participant characteristics also limited the depth of some research objectives; not all had used HRT, accessed menopause-related healthcare, or experienced menopause, which restricted the scope of questions but added diversity in perspectives.

The broad research focus may have diluted the study’s depth, and a narrower objective – such as exploring immigrant Muslim women’s views on HRT – might have been more beneficial. Additionally, women with negative menopause or healthcare experiences may have been more inclined to participate, possibly contributing to the predominance of critical perspectives. However, this aligns with previous research, lending credibility to our findings.

## Conclusion

This study highlights menopause as a profound transition for immigrant Muslim women, impacting multiple aspects of their lives, from physical and emotional wellbeing to workplace confidence. Participants felt unprepared for menopause due to limited knowledge and available information, which exacerbated the stress and fear associated with its symptoms. Cultural beliefs and social stigma around female ageing and the body influenced women’s understanding of menopause, often limiting their knowledge. Health-seeking behaviours varied: some women accepted menopause as a natural phase needing no intervention, while others actively sought treatment, making lifestyle adjustments such as diet and exercise. However, a lack of awareness about menopause-related healthcare options prevented some from accessing needed support. Diverging views on HRT reflected broader tensions between normalising menopause and medicalising it. Misconceptions and fears about HRT’s side effects created hesitancy, although those who tried it reported life-changing benefits. Overall, participants’ experiences with MRH were largely negative. Many women encountered dismissive healthcare professionals, limited specialist care, and time-constrained appointments, leaving them feeling unsupported and forced to manage symptoms alone. This reveals a critical gap in healthcare for Muslim women navigating menopause and underscores the need for more informed, culturally sensitive, and accessible menopause care. Despite challenges menopause was a time of new beginnings prompting positive change.

## Recommendations

### Practice and policy

We propose nationalised incorporation of matters of menopause in routine health-checks in order to adequately inform women on menopause and options for MRH. Addressing language barriers in healthcare through interpretation services is crucial to ensure effective communication and patient understanding.

To improve MRH, we advocate for policies requiring comprehensive menopause training for healthcare providers, particularly general practitioners, as recommended by the British Menopause Society and Women’s Health Concern. Training should cover symptom recognition, treatment options, and holistic self-care strategies, equipping providers to advise women confidently. Menopause should be recognised as an important life stage and an opportunity for preventive care to reduce long-term health risks.

### Education

Educational outreach is essential, especially for immigrant Muslim women, who may have less knowledge of menopause due to cultural silence on the topic. Accessible, culturally sensitive resources in multiple languages commonly spoken within the Muslim community should be developed and widely distributed through community centres, places of worship, and healthcare facilities. Currently available resources are challenging to access without prior knowledge of menopause, limiting their reach.

### Further research

Future studies should focus on specific groups (pre-menopausal, peri-menopausal, or post-menopausal) to gain deeper insights into each stage. Research focussing on an individual phenomenon such as only exploring immigrant Muslims women’s experience of MRH would allow for a more in-depth understanding. Additionally, quantitative studies with larger, diverse samples would help measure symptom severity and knowledge levels, incorporating women with varied education, employment, and migration backgrounds.

## Supplemental Material

Supplemental Material - A grounded theory study exploring immigrant Muslim women’s perceptions and experiences of menopause, hormone replacement therapy and menopause-related healthcare in the UK Supplemental Material for A grounded theory study exploring immigrant Muslim women’s perceptions and experiences of menopause, hormone replacement therapy and menopause-related healthcare in the UK by Reemanne Baghdadi, Neil Singh and Anne Gatuguta in Post Reproductive Health
