# Supplementary material for: A grounded theory study exploring immigrant Muslim women’s perceptions and experiences of menopause, hormone replacement therapy and menopause-related healthcare in the UK
Source: Post Reprod Health. 2025 Mar 2;31(1):33–8. doi: 10.1177/20533691251322823 (PMC11909765; doi:10.1177/20533691251322823)
Supplement: Supplemental Material - A grounded theory study exploring immigrant Muslim women’s perceptions and experiences of menopause, hormone replacement therapy and menopause-related healthcare in the UK [file sj-pdf-1-min-10.1177_20533691251322823.pdf]

Supplementary material

Participant characteristics table

Pseudonym	Age	Menopausal status	Has taken HRT?	Age at onset	Years in the UK	Level of education	Employment	Relationship	Children	Language of interview
Rania	50	Menopausal	Yes (Currently)	35	24	Higher education	Academia	Divorced	3	English
Maisie	42	Pre-menopausal	No	-	34	Higher education	Research scientist	Married	3	English
Pippa	56	Menopausal	No	50	43	Higher education	Nurse	Married	2	English
Hannie	50	Pre-menopausal	No	-	3 months	Higher education	Unemployed	Married	4	Arabic
Summer	52	Peri-menopausal	Yes (no longer on HRT)	49	25	Higher education	Unemployed	Married	3	English
Beth	51	Pre-menopausal	No	-	30	Higher education	Support worker	Married	3	English
Jamie	49	Peri-Menopausal	No	47	12	Higher education	Withheld	Married	3	English
Jess	60	Menopausal	No	53	8	High School	Housekeeper	Married	2	Arabic
Georgia	55	Menopausal	Yes (No longer on HRT)	45	50	Higher education	Enforcement officer	Single (Never married)	0	English
Keara	40	Pre-menopausal	No	-	37	Diploma	Healthcare professional	Married	1	English
Sasha	49	Pre-menopausal	No	-	44	Higher education	Nurse	Married	2	English
Lily	53	Peri-Menopausal	Yes (Currently)	45	40	Higher education	Social worker	Married	3	English

Table 1: Participant characteristics

Table 2 - Interview guide

Icebreaker – Thank you so much for taking part in my research! Just to remind you this interview is totally anonymous, so feel free to speak as openly as you are comfortable with. The purpose of this study is to understand your personal experience with menopause and menopause-related healthcare. Treat this like a little chat, there are no right answers, it's all about you. – You don't have to answer any questions you are uncomfortable with.	
**Question Category**	Question and prompts
**Experience of and knowledge of +attitudes towards menopause**	Tell me about what you think menopause is?<br>- Symptoms?<br>- Tell me about what you know about long-term risks related to menopause?
	What does menopause represent to you?<br>- Tell me about how menopause has affected you?<br>- Is menopause an accepted time in your life, why or why not?<br>- Does it change who you are as a woman?<br>- Has menopause effected your work life?
	- Tell me about how your community/Culture of origin view menopause?<br>- Does religion impact your view on menopause? Tell me about how religion affects how you view menopause?
**Menopause-related health seeking behaviours and selfcare**	Tell me about how you seek information and advice on menopause?<br>- Healthcare professionals?<br>- Family or friends?<br>- Internet?<br>How does information you've found on menopause help you?
	How do you cope with menopause and its symptoms?<br>- Do you know of any treatment?<br>- Do you have any selfcare methods?<br>- How did you learn of these treatments/methods?
**Knowledge of and attitudes towards HRT**	What do you know about hormone replacement therapy?<br>- What does it do?<br>- Short-term benefits?<br>- Long-term benefits?<br>- Risks?
	What do you think of hormone replacement therapy?<br>- Would you consider using hormone replacement therapy?<br>- Why/why not?
	Do you know enough about hormone replacement therapy to make informed choices?
	Tell me about what any other healthcare provider have discussed with you about menopause or menopause-related healthcare?
**Experience of menopause-related healthcare**	- GP?<br>- Nurse practitioner?

	Tell me about any menopause-related healthcare you have received?<br>- Are you happy with the healthcare you have received, why or why not? What do you need from your GP/healthcare provider and what can they do better?<br>- Would you feel more comfortable with a menopause specialist?
	Has your GP or any other healthcare provider provided you with information about hormone replacement therapy?<br>- Has this information equipped you with enough knowledge to make an informed decision?

Table 2: Semi-structured interview guide

Demographic of participants

**Parameter**
Age
Education
Employment
Years in the UK
Marital status
Menopausal status

Table 3: Participant demographics

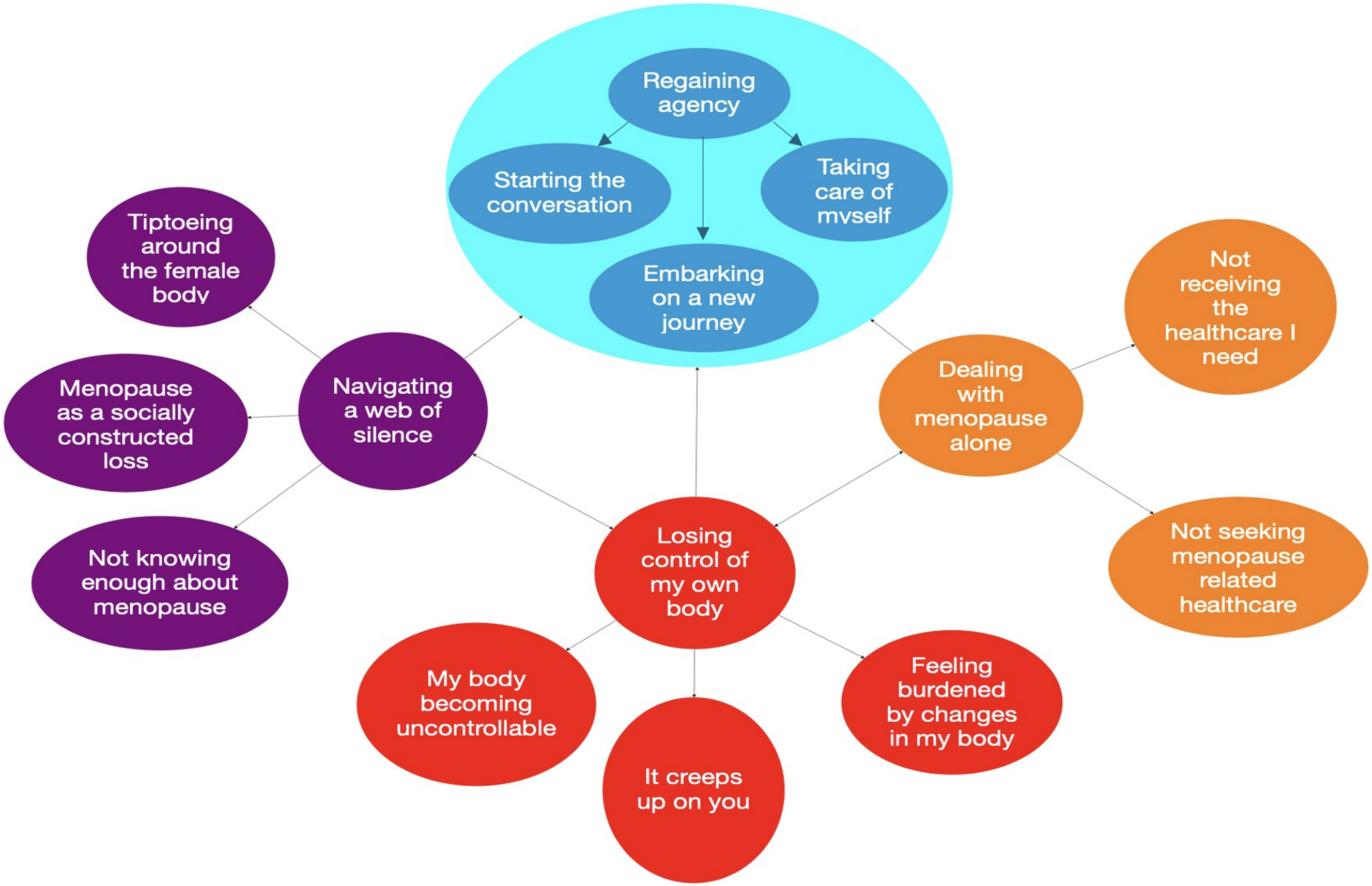

Figure 1: Category and sub-category relationship diagram

Category 1 data table

Subcategory 1.1: My body becoming uncontrollable		
**Focused codes**	**Open codes**	**Patient codes**
**Being affected by changes in appearance**	Changing appearance	"Your skin gets more drapey, gets drier, your hair goes very wirey." (Keara)
	Being unable to lose weight	"I've noticed that I've gained weight, which I find difficult to get rid of before it was easy for me to just go on a diet." (Jamie)
**Noticing sudden change**	Having no control over bodily changes	"That dial moves. And you just think, what? What's happened? What? Something's changing and you've got no control over it. You've just got to go with whichever way your body's taking you." (Lily)
	Losing control over body	"I think there is just something that's going on in your body that you have no control over." (Keara)
**Overwhelming physical symptoms**	Burning up	"Sometimes I feel like if I touch my arm when I'm having a hot flash, I would leave a burn mark of my fingers." (Georgia)
	Menstruating irregularly	"You could be bleeding for three weeks, stopped for a week start again it impacts on you very negatively." (Pippa)
**Subcategory 1.2: Burdened by changes**		
**Changes in cognition**	Being unable to concentrate	"My concentration is not like before…. So, when I'm reading or studying, I need to repeat things multiple time just to get that? Yeah, it's so hard to memorise things." (Jamie)
	Feeling frightened and vulnerable	"I was feeling really frightened, actually. I was feeling very frightened, memory is something that makes you feel older, it makes you feel more vulnerable." (Lily)
**Symptoms affecting work**	Being distracted at work	"You're very distracted and you can kind of like, you know, forget. You know, the thing is that you worry about what have I forgot." (Pippa)
	Being unable to deal with work demands	"I don't like how I'm feeling. I work full time, I work with young people, I work with 18 plus, young people that have left care… reflecting now and thinking about it, I felt really rough." (Lily)
**Mood dysregulation**	Feeling emotional	"I still have this like emotional, you know, like sudden feeling of sadness and you want to cry or you just sitting on edge anything will irritate you." (Summer)
	Suffering from depression	"I suffered from depression long term depression and I just I found it was really hard to you know carry on my life… I'm convinced my depression part of it is a side effect of my menopause." (Rania)
**Subcategory 1:3: It creeps up on you**		
**Confusion and unexpectedness**	Not conceptualising menopause	"You know, when I was younger and living my life like normal, I did not even expect a day like this would occur… I didn't think about it ever." (Jess)
	Feeling confused and disorientated	"It kind of creeps up on you. I think it can be confusing. I think it can be disorienting." (Pippa)
**Lack of certainty**	Being uncertain of menopausal status	"Nothing else has been set clear. It doesn't feel like anything's been made definitive by senior clinician. As where they go, actually it's been confirmed." (Keara)
	Feeling uncertainty about menopausal status	"Thought for me rather than still thinking, am I? aren't I? is this it? is this what it's like? I wanted someone to say yes it it's proven we've done a blood test, yes, you are now going through menopause." (Lily)
**Crossing that bridge when we get there**	Not receiving information from HCPs prior to menopausal	"Once I enter that phase, I would probably only know then, whether my GP or my sort of care providers around me are in a situation to give me the right advice." (Maisie)

	transition	
	Putting off learning about menopause	"I mean I guess cause I'm not going through it. I wouldn't be looking into it necessarily, probably if I started going through it, that's when I would look into it a bit more." (Keara)

Table 4: Category 1 Losing control of my body - focus, open and participant codes

Category 2 data table

**Subcategory 2.1: Not receiving the healthcare that I need**		
**Focused code**	Open code	Participant code
**Poor menopause related healthcare**	Feeling traumatised by healthcare encounter	"I went for an ablation of my, you know, thickened womb lining… I was thinking, you stupid man, I've been telling you to stop, I can't take the pain, I was so traumatised." (Pippa)
	Being left to suffer	"I feel really angry that I was left to suffer for almost 13 years. The solution and the help were there, but they just did not want to give it to me." (Rania)
	GP disregarding symptoms until it affected husband	"I remember speaking to one doctor and I just happened to say actually, I've lost my libido and I don't want to be a companion to my husband I want to be his wife… I've laughed about this thing since thinking it was a male GP and as soon as I said it's affected my sex drive and my husband he decided to check with a pharmacist." (Lily)
**Not heard by GP**	Feeling a lack of sympathy	"The doctors that I got just weren't sympathetic. I felt like I had to really explain how I was feeling, and I just felt like how they weren't getting it." (Lily)
	Feeling lack of sympathy	"The doctors that I got just weren't sympathetic. I felt like I had to really explain how I was feeling, and I just felt like how they weren't getting it." (Lily)
**Systematic issues within the healthcare system**	Perceiving a lack of specialism in women's health	"I think there needs to be a change in general practice it's definitely the infrastructure of training for the future doctors… even getting nurses specialising in Women's Health. Just to just offer a clinic, just to give some advice and support." (Keara)
	Perceiving a lack of training and resources	"Lack of training maybe because of lack of resources, maybe busy NHS." (Rania)
**Lack of standardisation among GPs**	Receiving good care after speaking to different GP	**"**Then they changed my GP, I have a good GP now, she listened to me and she tried HRT for me and it actually improved my symptoms a lot." (Rania)
	Receiving treatment after speaking to different GP	"She was the one that put me on the repeat prescription. But before then, every few months, I was literally going right battle time I need to get them to prescribe it for me." (Lily)
**Frustration over expectation to manage menopause alone**	Feeling frustrated with expectation to deal with it	"You know women for hundreds and hundreds of years, you know, dealt with it. Why can't you?... but that's natural. That's how it should be. Why are you complaining?" (Rania)
	Feeling frustrated with having to work it out alone	"A woman has just got to deal with it and just work it out on her own, and then the GP surgeries just go well, it's just a woman's thing." (Keara)
**Subcategory 2.2 Not seeking menopause-related healthcare**		
**I don't think I**	Letting	"I didn't do anything about it. I just let them be. I think. Initially when it

**needed menopause related health care**	symptoms be	happened, I just went with it." (Georgia)
	Believing symptoms weren't severe enough to require medical intervention	"No, I never did go to a doctor. Maybe because my symptoms weren't very severe, and it went by easily" (Jess)
**Taking HRT on my own terms**	Perceiving menopause as a natural occurrence	"I don't see it as something bad really. It's not an illness, it's not a disease… It's just a natural thing, so unless I find the symptoms really severe or uncomfortable, I'm not thinking about taking anything." (Beth)
	Symptoms not warranting HRT	"I've been thinking about it, but because I found the symptoms are not severe, they are not affecting me to that degree that I go and seek for HRT." (Jamie)
**Not knowing about treatment options**	Being unaware of HRT	"What is that (HRT)? I think I've heard of it, but I don't know anything about it." (Hannie)
	Being unaware of HRT	"No, I've never heard about that (HRT), what is it?" (Jess)
**Lack of awareness of the long-term risks of menopause**	Being unaware of long-term risks	"No, I had no idea that this could happen." (Jess)
	Knowing little about long term risks	"I do know some and like maybe the loss of bone mass and depression. That's about it. I think that's all I know about." (Rania)

Table 5: Category 2 - Dealing with menopause alone - focus, open and participant codes

Category 3 data table

**Subcategory** 3.1**: Not knowing enough about menopause**		
**Focused code**	Open code	Participant code
**Limited knowledge of menopause among participants**	Not being aware of what menopause entails	"There is a specific hormone… what is it called? Oh oestrogen. It has something to do with the period and fertility." (Hannie)
	Having limited knowledge of menopause	""I knew very little about the menopause. All I know is that when you go through the menopause, you go from being able to have children, to not being able to have children." (Georgia)
**Lack of education among community**	Community not being educated	"They're not educated enough about it." (Summer)
	Mother not being able to gain knowledge	"My parents were illiterate as well, so I guess whatever they learn, whatever my mum learned was through spoken learning, it was nothing that she read. Because she couldn't read or write any language." (Lily)
**Needing guidance**	Needing guidance from GP	"I think you know when you go and see a doctor, it's maybe not a bad idea to say, you know, just to make you aware." (Lily)
	Needing education from school	"Even at school I know you talk about sex education, but actually just the woman's cycle through life span, just so they kind of know in advance what. That they know at some point they're going through the menopause and what that will mean." (Keara)
**Subcategory** 3:2**: Tip-toeing around the female body**		
**Shame and stigma around**	Stigmatising women's	"All things women's health is taboo." (Jess)

**the female body**	health	
	Shaming women's bodies	"I had a miscarriage recently, and I was made to feel like I just couldn't talk about it, I was almost shamed. Women feel shamed for talking about their bodies and what goes on." (Keara)
**Menopause is personal**	Others not wanting to discuss menopause	"The issues of some females is that they don't really want their bosses to know when they're cycle is, and it's like ohh no, that's a personal thing…I don't want to have to tell people that I'm having my period or going through the menopause." (Pippa)
	Not wanting to tell others about her menopause	"It's a very, I think sensitive topic… I did not want people to know that I have menopause and I still till now I don't want people to know that I am you know I have menopause" (Rania)
**Culture hindering conversation**	Culture preventing conversation	"I guess it's another cultural thing. It's not spoken about, unfortunately." (Maisie)
	Not speaking about menopause due to culture	"Everything is hush hush. But then when you come from places like Pakistan and India back in the day nothing is spoken about." (Pippa)
**Negatively viewing discussion of menopause**	Negatively viewing conversation of menopause	"They're complaining and they're moaning and they're winging." (Maisie)
	Believing menopause is just something to talk about	"It seems to be when it happens to someone, it's like something to talk about. So, my friends or whatever, and they just start talking about it. "ohh, I've got the hot flushes, I've got this, I've got that."" (Beth)
**Subcategory** 3:3**: Menopause a socially constructed loss**		
**The use of negative terminology**	Using negative terminology	"They call it the age of the despair. So, people don't talk about it"(Rania)
	Perceiving menopause as age of despair	"People would talk about women whose periods stop as if that was it, as if you were finished, your life was done theoretically and practically, and you've reached the age of despair so your life will be full of despair upon despair and depression, and you'll even look old and tired." (Jess)
**Value of reproductivity**	Feeling like your turn is done	"They think when your reach menopause your turn is done." (Jamie)
	Culture placing high weighting on reproductivity	"I think our culture definitely belonging to the culture that we belong to, you belong to. I'm sure you understand that reproductivity has a high weighting, a very high weighting in our culture." (Maisie)

Table 6: Category 3 - Navigating a web of silence - focus, open and participant codes

Category 4 data table

**Subcategory 4:1: Embarking on a new journey**		
**Focused code**	Open code	Participant code
**Just another chapter of a women's life**	Perceiving menopause as part of aging	"I'm getting older, but that's the fact... You don't have to have menopause to tell yourself that you're getting older." (Beth)
	Feeling that menopause is the next stage	"So, it's a different stage. It's the next stage on." (Georgia)

**Menopause facilitating positive change**	Feeling wiser	"I feel the same or maybe more feminine now. It didn't affect me at all, no… because maybe I'm wiser" (Summer)
	Menopause opening doors to adventure	"I think it has freed me up to be more adventurous than to go travelling or do whatever I want… Free to be more like a man in a you just kind of get up and go. You don't have to worry about have you got everything that you might need? A change of clothing? You don't have to worry about any of that. So, it's great. Yeah, I feel good." (Georgia)
**Finding peace in religion**	Being able to pray and fast consistently	"I can fast now Ramadan with the people enjoy it and I don't have to do it later. And you know, I keep all my prayer" (Rania)
	Finding solace in religion	"You know sometimes I get a bit fed up… I deal with it and get rid of it. I distract myself, I go out, I remember God, I read Quran." (Jess)
**Separating self-identity from menopause**	Womanhood not being affected be menopause	"I don't see why it would do, unless you're still intending to have children. Then you're still a woman. I don't see how it changes your identity. It's just another phase in life." (Georgia)
	Separating identity from menopause	"I don't think it's affected my identity… I don't think it's going to change anything about who I am or what I'm doing." (Jamie)
**Subcategory 4:2: Taking care of myself**		
**Taking my healthcare into my own hands**	Pushing back against healthcare professionals	"I think that's what's made me recognise that I do really need to listen to my own body and actually really push back, push back with professionals." (Lily)
	Controlling healthcare encounters	"I try to control the interview; not cause I'm a controlling person. It's because I want them to hear me. Otherwise, you don't feel heard." (Pippa)
**Making changes for my health and wellbeing**	Increasing physical activity	"I try things at home sometimes just to go walking, do some activities, even if minor thing" (Jamie)
	Improving diet	"I just started to take vitamins and omega-3, eating healthy and until now alhamdulillah." (Jess)
**Unlike the women before me**	Previous generations being completely unaware of menopause	"She (Mother) wasn't aware of that she's going through hormonal changes in her body." (Summer)
	Being better equipped to seek information that previous generations	"Whereas people like myself and my sister's, we were able to educate ourselves or we know we can go online or we can speak to our doctors or whatever. And now I don't know what my mother did when she hit the menopause." (Lily)
**Subcategory 4:3: Starting the conversation**		
**Educating my children about women's health**	Having the conversation at home	"I discussed with them things that my mum never discussed with me, so as they're coming into puberty and stuff like that and learning about things at school, my thing is to make sure that we have a discussion at home." (Maisie)
	Ensuring sons are aware of periods	"When I was fasting and when you know my boys were growing up and everything, they would ask like mum why are you eating and it's like well you know when women have their period we don't have to fast. I did that on purpose to make them aware." (Pippa)
**Open discussion makes the**	Using humour	"I remember I was at work I got a hot flush I went outside and, on my way out I said to all my colleagues "menopause, hot flashing" (laughter) and they all started laughing, I wasn't embarrassed at all, there were

**transition easier**		even men there." (Jess)
	Creating acceptance through discussion	"When you talk with people, especially people who are in you at your age and you going through same changes this helped a lot actually. It gives you more like acceptance and understanding of what's going on and what the next step could be for you." (Jamie)
**Things are changing**	Women speaking out	"I do think it's starting to change. I think women are trying to just speak out, just be trying to encourage a change of behaviour." (Keara)
	Feeling a sense of solidarity through media representation	"I feel as though those people that we were watching on, you know, Davina used to present Big Brother years ago and things like that. It's like, ohh, right they're going through the same thing that I am and you feel as though you're going through that journey with them, which is quite nice." (Lily)

Table 7: Category 4 Regaining agency - focus, open and participant codes

Memos (appendix 1)

Reflective memo: Being a Muslim Female researcher, I intuitively understood what participant 2 (Maisie) meant when she said "Belonging to the culture we belong to, you belong to. I'm sure you understand that reproductivity has a high weighting". I didn't feel the need to probe further as I understand that culturally within the Muslim community childrearing is a major role for women. Probing further may have suggested that I was more of an outsider to her experience/not on the same wavelength.

Analytical memo: P6 had a very natural view of menopause and seemed to minimise the potential impacts – was this because she was pre-menopausal?

Analytical memo: Premenopausal participants appear to have very little knowledge of HRT. Does this lead to unpreparedness when they do reach menopause? Potentially as a result of lack of conversation and education, leading to confusion and unexpectedness.

Analytical memo: Participant 1 (Rania) negatively drew from example that women have dealt with menopause for hundreds of years without medical advice – Feeling that her menopausal experience was invalidated and dismissed. Whereas Participant 6 drew the exact same example but more positively explaining why she thinks that she does not need HRT.

Reflective memo: Interviews with P4 and P8 were translated by myself from Arabic – Was anything forced onto the data during translation? I will go through the translation to ensure it is as close to the original as possible. However, attempting to get direct translations from Arabic is problematic.

Sample interview transcript

Participant 12: Lily	
Question	Answer
So, tell me what you think menopause is.	Menopause is a period in a woman's life where her body goes through, I would say quite substantial change, having experienced it and when suddenly things change, actually, emotionally, and physically, because obviously your period stops. So, it has an impact on you physically like that biologically and I think emotionally as well. So, I suppose that's what menopause.<br>So, I didn't really you know, I used to hear about what the menopause was but I never ever really thought about it. Even when I started to get the symptoms. I didn't. I didn't relate it to being menopausal. I didn't make that connection at all. So, it was just a word. Before, it was just a word. Women get menopausal, yeah, you know, didn't understand it, didn't really think about it. It's something far off. And then it just kind of creeps upon you. For me, it felt like it just crept upon me. Yeah.
When you say it crept up on you, do you mean that you weren’t prepared or ready to go through menopause?	It wasn't something I even thought about. You know, you get your period. I started my period when I was 12 years old and it's just something really, you get your periods. I've never used to really have heavy periods, so I used to have a period for about 3 days and it's kind of medium to light. So, periods for me wasn't this big thing, I hated them, don't get me wrong, especially as a teenager. But I suppose as I got older and you have conversations with other people and you hear people really struggle with their periods, don't they? And I used to think ohh, that's not me mine are quite light. And then even when I got to the point where my period stopped, I didn't get heavy periods. They just did, just got lighter and lighter. So, I just kind of when I say a crept up onto me, do you know what I mean? It's not something. And I also, I mean I recognised I was a menopausal at the age of 47 and I always thought it was something that happened to older women, sort of in their 50s and onwards. You see, I knew you could get it sooner. Do you know what it really wasn't something I thought about for me, and there was nobody around me that was talking about it as well. Um, even at work I work in a team where we're mostly women out of a team of 14, my manager is male, and two of my colleagues were male. And we're all roughly the same age. So, we all kind of hit the menopause and then you’d start talking about it we started sharing our experiences, but before then, it wasn't something I really heard many women speaking about. I've got two older sisters they were still getting their periods. One is a year and a half older them me, and the other one is three years older than me, and they were still getting their periods like I think I might have hit the menopause before they did. It's not something we really spoke about. And my mother never spoke about it.
Why was it not something spoken about?	Growing up periods and things like that wasn't something that was spoken about. So, puberty anything like that, I mean, I know it sounds really daft, but I even when I got my period I remember didn't really know much about it, growing up in school in the 80s, in Lancashire, it wasn't something that was spoken about at school, I don't remember anything in like Junior School being spoken about. And then at the age of 12, I got my period and my mum never spoke about it. And I remember even things like when we got our bras, you know, when you washed them you. The kind of made sure the males in the family never saw your underwear. If you hung your washing, your bras and knickers were always hung under an item of clothing. So it was never visible. So even anything to do with the female body was just not spoken about. You know, it was just one of those things. And we found it was quite a common thing with the Asian women and. Yeah. And I think I think its cause periods and things like that were considered you were unclean. Growing up as a Muslim you weren't allowed to pray when you're on your period. It was something you kept quiet about, so you didn't want to tell anyone that you're on your period because then so you, which is why when we got to puberty, about 12,13 we stopped going to mosque because obviously you can't touch the Quran. You can't pray. And it's not something that you tell people that you're on your period. And obviously if you didn't go to mosque for a week. It meant that ohh she's on her period. So, it's all very hush hush.

So, tell me about the symptoms or changes that you experienced?	I've always been a really good sleeper and I've always loved sleeping. It's one of my favourite things. I love sleeping and I never have trouble going to sleep as well. So, I noticed I was sleeping a lot lighter. Again, I didn't link it with the menopause, I just thought ohh you know it was one of those things like ohh I I'm waking up over the smallest of things and why is it taking me so long to get to sleep and then everyday about 4:00 in the morning. I'd wake up and I think what's going on. Why am I waking up? So that was one of the first things I noticed. But didn't make the connection. I just thought my sleeping patterns changed. Um, I notice I was getting warmer. My body was feeling warmer.<br><br>It was only when I was on a train with my daughter, so we were coming back from London to Brighton and I noticed I was sweating more on the face and I was talking to my daughter, we were sat on the train with a table in between us talking to her and she just said mum oh my God, I can just see sweat just building on your upper lip and. I said, I know I can feel it and she said, but I can see it in your face was dry and now you've just got literally beads of sweat on your face. That's when I thought. Something is not right. You know, it was kind of one of those where she commented on it. Even then, I didn't go to the doctors. I just thought I'm getting a bit warm. I think at work I said I'm feeling really warm and maybe somebody sort of said you know maybe its menopause. I was thinking I'm sort of you know only 47 I didn't think of it as a major thing that I needed to rush to the doctor's for, but I think I started to feel more tired because I wasn't sleeping well, so it was having an impact on my day.<br><br>The heat in my body, it was like a furnace, and it would start on my back and I would be literally stood in the doorway. I'd be rushing out the kitchen or the living room, flinging open the doors, the front door, any door and just standing there thinking I need a cold blast to hit me. And then that's when I thought, right, I need to do something. And I had a chat with some people at work and ironically, then the City Council had sent an e-mail out, because I work for the council, saying that 65% of the workforce were female over the age of 40, so they were going start introducing menopause workshops for managers, males, females to for everyone to go to so they understand what the menopause is because they're recognise that actually their workforce was getting older and a high percentage were of a certain age. Then I went along to that because I thought I need help; I need help and I didn't know what sort of help I needed to get. So yeah, then I went to the doctors and decided I needed help.
These workshops that the Council put on, did you think they were helpful?	I didn't want feel anxious. I didn't want to feel tired all the time. I didn't want to feel irritable. I wanted to have a good night's sleep. I wanted to feel more confident within myself, because I'm fairly confident person, I think. Anxiety's never been a real issue for me, and suddenly that was a thing. So, all of these things were happening. I remember thinking, no, I want to feel like I felt before. I'm so I was kind of just trying to absorb any and all information because I didn't know anything about menopause.<br>All of my memory, my memory was going and that was really frightening me. Actually. I found that very frightening and a colleague at work, Hillary, whose 10 years older than me, said to me that when what she does and what she did was carry a notepad and pen around with her all the time so she could, you know, just write things down because she knew she'd forget them. And I do it got to a point where my children would say, but I've had that conversation with you. And I'd be saying I don't remember. And they're that sort of make jokes but inside I was feeling really frightened, actually. I was feeling very frightened, memory is something that makes you feel older, it makes you feel more vulnerable. When talking to people and you thinking you've told me this and I can't remember and then suddenly forget simple words that you just knew which I still do by the way. You know, there's so many things I just forget.

Are you still transitioning, or do you think you are postmenopausal now?	Well, it's interesting and that's the conversation I've had with a few people because I'm a bit unsure. I remember saying to the doctor, cause I so I take HRT gel. So, I started off on the patches. They kept falling off, and I remember thinking, I want to feel better and the patches. I kind of wanted, um, everything that was feeling to go away and with the patch as it didn't. So, I remember going and the doctor tried me on for three months. And Then I went back after three months, I was desperate, thinking why don't I feeling any different? Sorry, I don't know. What? See, I've forgotten what you asked me. What did you ask me?<br><br>Ohh yes yes I am. I still get I still get hot flushes, so even now I think to myself, why am I feeling hot? My husband loves putting the log burner on and we continue to have arguments cause he'll say it's cold and I'll be saying no, no, it's hot, it's hot. Please, you know. So no, I still feel as though I'm going through it and I'm a bit remember saying to the doctor, how do I know when I'm come out of it? And she said, well, you'll just know so. I, to be honest, I don't know, but I still have moments where I just I could feel my body warming up for no reason. So, then I think to myself right, it does that mean I'm post-menopausal?
Are you happy with the healthcare you've received for menopause	No, no, it wasn't good, to be honest. Well, I was seeing a doctor's that just seemed a bit disinterested. I remember saying, do I need to have a blood test to see whether I'm in the menopause? Because I'd heard you could get a blood test, so they know for certain. And they said, well, if you're feeling these symptoms, then you are so thought well, ohh okay okay so I'm menopausal then, they went well if you're saying you've got this, this and this and you are. I kind of wanted someone to say yes you are. It's all kind of quite, I don't know, It just kind of comes on and things change for you and quite a lot of things. I remember reading the symptoms and like basically so many things change. I just kind of I don't know. . I thought for me rather than still thinking, am I? aren't I? is this it? is this what it's like? I wanted someone to say yes it it's proven we've done a blood test, yes, you are now going through menopause. I think it might have been easy for me because I think I could have made, I suppose, you know when you know someone says, right, you've got this and you need to do this then it's a bit easier rather than that uncertainty.<br><br>The workshop I went on, the lady that was delivering it, she said that she was on testosterone because that's one of the things that women lose testosterone and she said it makes you feel great and you know how I said I just wanted to feel good again. I remember just latching on to that thinking. Right. I need to try and get testosterone. And my God, it was so, so difficult. I felt the GP was very.. So when the patches didn't work and there were, all, you got wait three months out. Thinking I feel rubbish and you want me to wait three months. I've done it for a month and I'm telling you, I don't feel any different. Then they said right now take the tablets and that took the tablets, and then I was still thinking I don't feel any different. So, then another three months of that and again I'm feeling pretty rubbish. Then eventually they said to me right, you know, there's the gel, but I'd have to have the Marina coil fitted. So, then I was just like, you know what? Whatever I need to do, I'll just have to do that because I don't like how I'm feeling. I work full time, I work with young people, I work with 18 plus, young people that have left care, children that have left the care system. So they've got very complex issues, they're quite chaotic, there's an awful lot going on. So it's one of those jobs where you're chasing young people up and you know, can get quite stressful because they lose their accommodation and they're sleeping on the streets and you've got to try and find housing for them and stuff. So, you know, it's one of those jobs where you just don't know what's happening, and then you're chasing stuff to try and sort things out for young people. I just felt like I needed something, reflecting now and thinking about it, I felt really rough. Then when I wanted the testosterone, I remember two or three GPS I spoke to said we've never prescribed testosterone for a woman before. All the GPs that were getting were male. So be always like a telephone, "Ohh We've

	never prescribed it for women before", and I'd say, "but I know you can because I've been to this workshop and I've Googled it and you can". Then they were like, not sure, not sure. Then I remember speaking to one doctor and I just happened to say actually, I've lost my libido and I don't want to be a companion to my husband I want to be his wife. And as soon as I said that, I don't know where that came from I felt desperate. He just went ohh so it's affected your libido and I said yeah and ok. Well, now that you've said that I can check with a pharmacist colleague because I've never done it before, but you said it's affected your libido. I will check and find out what we can prescribe for you. I just remember thinking, well, if I'd known I just had to say that and I thought. I've laughed about this thing since thinking it was a male GP and as soon as I said it's affected my sex drive and my husband he decided to check with a pharmacist. Then he got back to me and said we can prescribe it for you. I really noticed a real positive difference when I when I got that, I felt more confident I felt more alive. But They didn't put me on repeat then when it finished or was about to finish, I remember ringing up and then I went through the same rigmarole because it lasted about three months. The same rigmarole of kind of going, but previous doctor prescribed it, and I aI feel so much better with it, can I not have it? They'd be like we've never prescribed it for a woman, ohh should they have prescribed it and all of this? Then I used to kind of go well, they have so please. It was very much had become not begging but really kind of going please can you you've already done it, there's nothing wrong with me, I'm happy with it. So, I always felt like I was always like psyching myself up for that battle for them to prescribe it for me again. Then on one occasion I rang up and I and they said, you know, when the receptionist says, why are you calling? And I said, well, look, I really need this. They won't put it on repeat for me, but I need this. She said, Ohh well, we've got a doctor in, we tend to refer the menopausal patients to her. It's like it's like a specialism or an interest of hers. So then she rang me, and honestly she just went, absolutely fine I'm going put it on repeat for you, not a problem. She was the one that put me on the repeat prescription. But before then, every few months, I was literally going right battle I need to get them to prescribe it for me. It is shocking and shocking that had to mention that, you know, like you know, it's like weird. And I and I've said, I've said to friends since, you know, if you want it, it worked for me when I said this. So you say yourself just say, you know, that's how it's affecting me and my husband.
Has menopause affected your identity in anyway?	Yeah, I guess it absolutely did have an impact. I think at the time I remember this; I think it's October 2017 when I had that that bit where I thought Oh my God I'm sweating and I constantly sweating where I was wiping things off and yeah, it just did affect my identity. I remember going to a wedding and we are sitting at this table and you know how you seated with people you don't know and stuff. I was chatting to this guy and it's quite noisy and I was leaning forward, and I just remember sweating and with the tissue literally doing this the whole time and at one point he said to me, I'm sorry, am I spitting at you? And I said no, no, no, no, no. I said I'm sorry I'm going through the menopause and I'm just sweating and it just felt, you know horrible, you know, so in identity in the sense where I was confident person and then suddenly, I thought Oh my God, I can't even speak to anyone without patting myself. And you do you feel sweaty all the time. You're putting on weight. I could lose weight so quickly, my weight gain is just here. It affects your confidence if that affects yourself, esteem for sure. So in that sense, is that what you mean by identity? How I felt as a person. Certainly, as a woman. I don't feel as attractive anymore. Ohh then I mean it's been what, five years now? Well, so I don't feel as attractive anymore and noticed my hair became coarser. God's just it just all goes down. Ohh it goes all goes downhill. My hair started to feel coarser. Reflecting at the time I was just going through it and then in in August, my son fell really, really ill as well, so I wasn't able to give that much time to thinking about what was happening with me because there was no, my son fell seriously. All so we were kind of managing his illness. So then he started to get better in 2019.<br>.<br>Then I started to reflect on it all because I kind of recognise I was in the menopause in October 2017, that was me kind of trying to find out

	what type of HRT worked for me, then getting the Marina coil fitted that just took forever.. Yeah. And then my son fell really ill. And then I think when he kind of started to get better, I started to reflect on the menopause and thought actually I've had a pretty rough time with the menopause. But there's some reflecting back there was actually a pretty rough time trying to keep it full time job going, sitting in the hospital again with my son. You know, the whole time, all of us, the whole family was affected and then go through the menopause. Cause I think I got quite a lot of the symptoms and things. So it really does affect your you affect you in so many ways. I think the biggest thing for me is the memory forgetting things and not being able to retain anything. So it's not just for getting things, it's that it's also trouble with retaining information.
You said you didn't know much about menopause and it was barely a thought to you. What would have increased your knowledge? What would have made you feel more confident or adequately prepared to reach menopause?	Also really good question, what would have helped? Think it's when you're not, when it's not happening to you, it's not kind of up there as a thing is. It's that with most things it's not on your radar. You know, it's going happen but it's not a priority in your life at that moment in time. I was getting my monthly periods. You know, life was ticking along. What could I do? Thinking what could help me actually. I don't know. I don't know cause I'm thinking even if they're so leaflet, I mean, would I have gone for the workshops if I was 35 years old? Yeah. Well, I wouldn’t have thought it was a priority for me. Do you know what I mean? So the Council was offering these workshops to everyone. I don't think it would have gone. I'll probably gone ohh menopause workshops but then thinking ohh well you know, doesn't happen to people when they are much older. Like I thought in the 50s. So even though so I don't know whether I would have to be honest.
I have heard that people have had health checks and they kind of check your weight and your height and ask about your drinking and smoking habits,. Do you think it would have been helpful if a GP mentioned menopause during that health check?	That rings a bell that rings a bell. No, I don't think they asked about menopause. I think it was just like just checking you over, I don't think so like I mean it was God, you know, I forget I forgot what I have for lunch yesterday, but never mind 13 years ago. But that does ring a bell.. But I think you're right. I think when you get to about 40. I think you know when you go and see a doctor, it's maybe not a bad idea to say, you know, just to make you aware that probably won’t t happened now. But these are some of the symptoms. Do you want me to give you a leaflet? Might not happen now, but just so you're aware. You know, or something like that or maybe 40 onwards they can just. Maybe sending just like a generic e-mail can be sent out, you know, just about menopausal symptoms just so you're aware rather than. I think with Asian families it's different, so my mother's generation things like sex, sex talk, puberty all of those things there were not really spoken about. It it's not something that you speak about. Like I said, you know anything like we couldn't even you know you hang your washing out and even now my daughter's my daughters hang their stuff out and sometimes I look I think Oh my God they've got it on display on the washing line. Then I think no, now times have changed, so even now I kind of have that feeling of should I cover it? Should I hide it because the dad or a brother might be walking around? So you know, it definitely wasn't anything that people speak about. They'll just quietly say, ohh my period are on and normally only ever knew was when they didn't, didn't get up to pray. You kind of thought ohh they're on their period. Unless you are very close, and you spoke to a sister or cousin or a family member. But otherwise it was so the praying was a big one, cause we pray five times a day. And because you don't want the male people in the family to know that that one week, 8 days whatever you're not fasting so they're pretend that they will sneakily have a glass of water, a cup of tea or whatever. So the male members of the family don't know that actually it's your time of the month. So anyway, sorry, I'm just kind of going off on one, but that that's how we grew up anyway. Very, very traditional like that.

	But you know, I guess it's different as well because my generation that fluently speaking English because my parents were illiterate as well, so I guess whatever they learn, whatever my mum learned was through spoken learning, it was nothing that she read. She because she couldn't read or write any language. But also you know there's that confusion between religion and culture. And is it religion they're following? Is it culture? You know, everything becomes very diluted, isn't it? They just take whatever they hear is, as you know. So we weren't allowed to bathe for eight days our period. So when your period started, we were told you not to bathe until the 8th day when you cleanse yourself. So, we weren't allowed to do that. So, but obviously, as soon as you start to think for yourself and you started to but initially for years you we didn't so when your period starts for eight days on the 7th or 8th day then you can cleanse yourself have a shower. Yeah. So, I don't know where that comes from. Whereas people like myself and my sister's, we were able to educate ourselves or we know we can go online or we can speak to our doctors or whatever. And now I don't know what my mother did when she hit the menopause. I don't know whether it's the same in your culture, but you know you have like, heating food and cooling food. Do you do you know about that? You know, in certain cultures? So, I know that a lot of our women in that certain generation in order to manage the body heat would certainly eat foods that are more cooling for the body.
Did you try any cooling foods?	I didn't. I just went Oh my God, I feel horrible. I just give me drugs. Give me anything you know. So no I didn't try that because I don't think I had the patience. Alright, I just felt rotten. I felt tired. I felt like I wasn't myself at all, so I just wanted a quick fix. You know, and I knew I could get a quick fix by going to the GP. If I lived somewhere like my extended family do in Pakistan. It might be different. Would have been using other things I would have been maybe using certain spices and certain types of food to try and regulate my body temperature, yeah.
So what about any other self-care methods?	I started doing yoga. I also noticed like physical things in my knees would ache a lot more. My hips would ache a lot more, so he got me into doing yoga, which I swear by it is fantastic. It really helped. I am more conscious of what I eat and I'm more conscious of what my body tells, and I'm sort of listen to my body a lot more. Because before I wasn't associating my symptoms to the menopause. It's a weird thing. You kind of feel young and whatever and alright with your life and then suddenly it's like a windscreen effect. Suddenly you go from here to kind of it moves. That dial moves. And you just think, what? What's happened? What? Something's changing and you've got no control over it. You've just got to go with whichever way your body's taking you. Some. I know some of my colleagues are kind of gone I haven't really noticed it. And then you've got something like me. I'm kind of going. Oh my God, I noticed all these changes.<br>And you've got no control over it and it's frightening. I felt frightened. I felt frightened by it.<br><br>So am I fully back? Not at all. Like I said, I still get some of the symptoms.<br>My anxiety is settled itself. It's fine, which I'm really pleased about. And one of the first things I noticed was I was able to sleep better, which was amazing because I can get through my day if I can sleep. It's a biggie if you're not if you're not sleeping it impacts on so much of your physiology and just mentally and everything.
I really wonder if there is anyway women can prepare themselves so it doesn't affect them so much or even be better equipped to	I think that's quite hard. I would much not quite hard because women get it at different stages. So how do you prepare for something when I get at 47, somebody might get it at 41, for example, or somebody gets it at 53, you know? So how do you prepare for something like that? Also I guess it's because it can be quite different for different people. What's good is that it is openly spoken about.<br>Which is fantastic. So in our office, honestly, we'll just go Oh my God, I'm feeling menopausal Bob. You know, it's like Whatever Yes, it's like it's like they've got this benchmark, but you could be here, but I'm and have all the symptoms. I totally agree with you, but because you haven't hit that benchmark, you're my manager just going have to accept it. This is how I'm feeling and we'll laugh and go. Well, you've just got a bunch of

manage it	like 40–50-year-olds that you're managing, Bob. So, and we're all menopausal. And he just laughs it off, you know, because what more can he say? There's lots of podcasts about it. I think Davina McCall and all these people, it's almost and they're kind of my age. I think they're in their early 50s and stuff aren't they? I feel as though those people that we were watching on, you know, Davina used to present Big Brother years ago and things like that. It's like, ohh, right they're going through the same thing that I am and you feel as though you're going through that journey with them, which is quite nice. It's not embarrassing to talk about it. I've gone into meetings where I'm sitting with like strangers. Men or females, and I'll just go sorry. I'm going through the menopause. So you're just going have to repeat that because my minds just gone and I'm not embarrassed to say that, which is quite nice. And I think it's because I hear it's spoken about on the television. I've read it about it. You know, it's out there. So, you can I feel as though I can say quite openly, you know, I've said some stupid things, and somebody will go. What did you say? I'll say sorry, I'm menopausal. I've come out with something stupid so I almost kind of use it as a bit of a, it's my little protective mechanism now. I'll just say ohh I'm having a menopausal moment.
	Yes,it's like it's like they've got this benchmark, but you could be here, but I'm and have all the symptoms. But because you haven't hit that benchmark, you're OK. That's normal. Well, it's not normal. What is normal? But you know, yeah, you're right. It's like they've got it. Its proper tick box exercise, isn't it? You get that number then you're this and if you haven't reached that number, then you're not.<br><br>You know, depending on who you get, you know. The doctors that I got just weren't sympathetic. I felt like I had to really explain how I was feeling, and I just felt like how they weren't getting it. Like they didn't know what to, just giving me the stock, OK, well, we can put you on this, that and the other you've got to stay on it for three months, though. And even when I was saying I, I don't think it's working and I think that's what's made me recognise that I do really need to listen to my own body and actually really push back, push back with professionals. But I think that takes a certain level of, I guess, type of person that you are. You know, certain levels of confidence to sort of be able to push back. My mother wouldn't have been able to do that, you know. You know if you're a traditional woman that doesn't speak the language, it's not, you know, your first language. You're not going to push back.
